# Rapid Mental Stress Evaluation Based on Non-Invasive, Wearable Cortisol Detection with the Self-Assembly of Nanomagnetic Beads

**DOI:** 10.3390/bios15030140

**Published:** 2025-02-23

**Authors:** Junjie Li, Qian Chen, Weixia Li, Shuang Li, Cherie S. Tan, Shuai Ma, Shike Hou, Bin Fan, Zetao Chen

**Affiliations:** 1 Key Laboratory of Medical Rescue Technology and Equipment of Ministry of Emergency Management, School of Disaster and Emergency Medicine, Tianjin University, Tianjin 300072, China; junjie@tju.edu.cn (J.L.); 2023246186@tju.edu.cn (Q.C.); li0606_@tju.edu.cn (W.L.); mashuai2019@tju.edu.cn (S.M.); houshike@tju.edu.cn (S.H.); 176267@tju.edu.cn (B.F.); 2 Medical College, Tianjin University, Tianjin 300072, China; lishuangv@tju.edu.cn (S.L.); cherie.tan@tju.edu.cn (C.S.T.); 3 Academy of Medical Engineering and Translational Medicine, Tianjin University, Tianjin 300072, China

**Keywords:** emergency medical rescue, mental stress evaluation, nanomagnetic beads, reverse iontophoresis, biosensor

## Abstract

The rapid and timely evaluation of the mental health of emergency rescuers can effectively improve the quality of emergency rescues. However, biosensors for mental health evaluation are now facing challenges, such as the rapid and portable detection of multiple mental biomarkers. In this study, a non-invasive, flexible, wearable electrochemical biosensor was constructed based on the self-assembly of nanomagnetic beads for the rapid detection of cortisol in interstitial fluid (ISF) to assess the mental stress of emergency rescuers. Based on a one-step reduction, gold nanoparticles (AuNPs) were functionally modified on a screen-printed electrode to improve the detection of electrochemical properties. Afterwards, nanocomposites of MXene and multi-wall carbon nanotubes were coated onto the AuNPs layer through a physical deposition to enhance the electron transfer rate. The carboxylated nanomagnetic beads immobilized with a cortisol antibody were treated as sensing elements for the specific recognition of the mental stress marker, cortisol. With the rapid attraction of magnets to nanomagnetic beads, the sensing element can be rapidly replaced on the electrode uniformly, which can lead to extreme improvements in detection efficiency. The detected linear response to cortisol was 0–32 ng/mL. With the integrated reverse iontophoresis technique on a flexible printed circuit board, the ISF can be extracted non-invasively for wearable cortisol detection. The stimulating current was set to be under 1 mA for the extraction, which was within the safe and acceptable range for human bodies. Therefore, based on the positive correlation between cortisol concentration and mental stress, the mental stress of emergency rescuers can be evaluated, which will provide feedback on the psychological statuses of rescuers and effectively improve rescuer safety and rescue efficiency.

## 1. Introduction

During emergency rescues, the mental health of rescuers can be adversely affected by many factors, including adverse circumstances, the urgency of tasks, and unknown dangers [1]. People who face prolonged exposure to external stimuli can become highly tense, resulting in physical fatigue, dizziness, and anxiety [2]. This ultimately results in an inability to continue participating in rescue efforts and even to face danger. The underlying mechanisms of these stress phenomena are that when individuals are stimulated by external factors, their mental stress levels increase. Excessive mental stress can lead to the disruption of the internal environmental balance in the human body [3,4]. Currently, the gold standard stress criterion is the State-Trait Anxiety Inventory (STAI). However, the STAI is subjective and its results are delayed, which makes it unsuitable for use in emergency rescue [5]. In comparison to scale methods, the assessment of mental stress based on physiological parameters is more objective and efficient.

In order to maintain internal homeostasis, the body will regulate the secretion and release of glucocorticoid via the hypothalamic–pituitary–adrenal (HPA) axis and sympathetic nervous system (SNS) [6]. Cortisol is the primary glucocorticoid released in response to mental and physiological stress, and it plays a crucial role in the body’s stress response and regulation [7]. Elizaveta found that fluctuations in cortisol levels corresponded with changes in people’s subjective stress levels, indicating that changes in cortisol concentration can be effectively used to analyze variations in stress [8]. It was discovered during Sarah Damaske’s research that workers with higher mental stress levels also had higher concentrations of cortisol [9]. Meanwhile, it was found that cortisol levels increase when individuals are exposed to external stimuli, and this regulates the cardiovascular and metabolic functions [10]. In conducting the Trier Social Stress Tests (TSST), Heather C. Abercrombie found that as stress levels increased, cortisol levels rose correspondingly, indicating that an increase in cortisol serves an emotional protective function [11]. These studies have demonstrated that cortisol can be employed as a biomarker of mental stress. Many studies have found that cortisol is present in various bodily fluids, such as interstitial fluid (ISF), in which the concentration of cortisol is positively correlated with that in plasma [12,13,14]. Therefore, the detection of cortisol in ISF can be utilized for the evaluation of mental stress.

Traditionally, approaches for cortisol detection have included electrochemiluminescence (ECL) [15], high-performance liquid chromatography (HPLC) [16], enzyme-linked immunosorbent assays (ELISAs) [17], surface plasma resonance (SPR) [18], radioimmunoassays (RIAs) [19], etc. However, all these methods require large instruments and take a long period of time to perform the accurate detection of cortisol, which makes them unsuitable for emergency applications. Therefore, compared to traditional methods, electrochemical biosensors are widely used for portable, real-time, and rapid detection applications, offering a convenient way to conduct health evaluations [20]. Moreover, based on substrates like hydrogel [21], polyimide (PI) [22], and others, wearable electrochemical biosensors exhibit excellent flexibility and biocompatibility properties [23,24]. For the wearable monitoring of cortisol, a reverse iontophoresis (RI) technique was utilized for the noninvasive extraction of ISF, and it is accessible and has a low risk of infection [25,26]. Given that the skin is the largest organ in the human body, it follows that the ISF can be obtained from a multitude of sites without compromising accuracy and convenience [27,28]. Therefore, with a non-invasive, wearable biosensor, cortisol can be monitored in emergency rescuers at any time and place, without affecting their normal work.

However, the stable immobilization and replacement of sensing elements onto electrodes remains a challenge, and this not only limits the flexibility of these biosensors but also increases their manufacture costs. Usually, sensors are discarded after completing a detection, which means many sensors need to be reserved in scenarios where multiple detections are required. This conflicts with the requirements for being portable and lightweight as a component of emergency rescue equipment in emergency rescue. Secondly, the analytes to be detected in emergency rescue may change at any time. For example, when a rescuer is injured, the analytes need to be changed from cortisol to uric acid to detect infection in a wound. Therefore, it is important to utilize electrodes with different sensing elements to detect different analytes through the same sensing platform. Usually, immobilization approaches for sensing elements are membrane entrapment, physical adsorption, and covalent bonding, and these present challenges in replacing the sensing elements. Thus, finding a substance that can not only bind proteins but also solve the elution issues is the key to replacing sensing elements. Nanomagnetic beads can effectively adsorb to magnets, and they can also immobilize antibodies through carboxylation modification [29,30]. Therefore, under the effects of an external magnetic field, nanomagnetic beads can be easily immobilized and removed from the surface of an electrode [31,32]. That is, with the application of nanomagnetic beads, the stable immobilization and replacement of sensing elements can be achieved.

In this study, we proposed a wearable electrochemical sensing platform for the detection of ISF cortisol and the evaluation of mental stress. Based on functional modifications to gold nanoparticles (AuNPs), nanocomposites of MXene, and multi-wall carbon nanotubes (MWCNTs), the electrode showed improved electrochemical properties. With the binding of a cortisol antibody to carboxylated nanomagnetic beads, the rapid immobilization and replacement of sensing elements could be controlled through external magnets. To achieve wearable application, a flexible printed circuit board (FPCB) was designed and developed with differential pulse voltammetry (DPV) and RI techniques, and this could be utilized for the non-invasive extraction of ISF and the rapid electrochemical detection of cortisol. Therefore, with this nanomagnetic-beads-based, non-invasive cortisol detection platform, the sensing elements could be replaced rapidly and the mental stress of rescuers could be evaluated effectively, providing feedback on the psychological statuses of emergency rescuers and improving rescuer safety and rescue efficiency.

## 2. Materials and Methods

### 2.1. Chemicals and Materials

The Ti_3_C_2_T_x_ MXene monolayer dispersion, MWCNTs water dispersion (10 wt%), and carboxylated magnetic beads were purchased from XFNANO Materials Co., Ltd. (Nanjing, China). The purified mouse anti-cortisol was purchased from Yuning Biotechnology Co., Ltd. (Shanghai, China). The cortisol was purchased from Diper Biotechnology Co., Ltd. (Shanghai, China). The MES buffer solution was purchased from Source Leaf Biotechnology Co., Ltd. (shanghai, China). The bovine serum albumin (BSA) was purchased from Budweiser Technology Co., Ltd. (Beijing, China). The chloroauric acid (HAuCl_4_), potassium ferricyanide (K_3_[Fe(CN_6_)]), and potassium ferrocyanide (K_4_[Fe(CN_6_)]) were purchased from Sinopharm Group Reagent Co., Ltd. (Shanghai, China). The phosphate buffer saline (PBS), lactic acid (LA), urea, glucose (Glu), uric acid (UA), and Nafion were purchased from Wokai Chemical Reagent Co., Ltd. (Shanghai, China). The 1-(3-Dimethylaminopropyl)-3-ethylcarbodiimide hydro (EDC) and n-hydroxysulfonic succinimide (NHS) were purchased from Macklin Biochemical (Shanghai, China). The low-resistance conductive carbon ink and Ag/AgCl were purchased from Tongbai Heyuan Trading Co., Ltd. (Henan, China). The polyimide film (PI) was purchased from Beilong Electronics Co., Ltd. (Guangzhou, China).

### 2.2. Instrumentations

Electrochemical approach cyclic voltammetry (CV) was utilized for the characterization of the electrodes, such as whether the functional nanomaterials and sensitive antibodies were successfully modified onto the electrode. The CVs were carried out with 20 mM [Fe(CN)_6_]^3−/4−^ as a redox couple, and the scan potential range was set at −0.4 V to 0.6 V. The AuNPs were deposited by using square wave voltammetry (SWV), with a scanning potential range of −0.8 V to −0.2 V. Cortisol was detected by DPV, with a scanning potential range of −0.6 V to 0.3 V. The morphological characterization of electrodes was performed by a Sigma 500 scanning electron microscope (SEM) from ZEISS (Germany). The concentration gradients of the cortisol were set to 0 ng/mL, 1 ng/mL, 2 ng/mL, 4 ng/mL, 8 ng/mL, and 16 ng/mL. All experiments were conducted at room temperature.

### 2.3. Fabrication of the Screen-Printed Electrodes

The electrodes of electrochemical sensor were fabricated through screen-printing technology. The counter electrode (CE) and working electrode (WE) of the screen-printed electrodes (SPE) were printed with low-resistance conductive carbon ink, and the reference electrode (RE) was printed with Ag/AgCl. A template for the electrodes was designed based on the modeling software, Adobe Illustrator 2022. The template size was 350 mm long and 250 mm wide on the outside by 300 mm long and 200 mm wide on the inside, and the mesh density was 250 (Figure S1a). Afterwards, the low-resistance conductive carbon ink was screen-printed onto the PI film as CE and WE. After being dried for 3 h, the RE was screen-printed using an Ag/AgCl paste.

### 2.4. Integration of the Reverse Iontophoresis Technique

A reverse iontophoresis technique was utilized for the extraction of ISF, from which the cortisol would be monitored. The extraction electrode was designed and fabricated on an 0.11 mm thickness PI substrate with a size of 17.3 mm wide by 52.7 mm long (Figure S1b,c). Then, a copper foil was fixed on the substrate by electroplating, forming a circle with a radius of 2.5 mm and a thickness of 1 μm. This circle was treated as a cathode. Afterwards, a copper foil circle of the same size was fixed near the anode and treated as an anode, forming a current circuit for the RI extraction of ISF. To ensure the extracted ISF could pass smoothly through the extraction electrode and reach the working electrode, micro-holes with radii of 0.38 mm were drilled in the cathode. Then, an adjustable current ranging from 0.5 mA to 1 mA was applied the extraction electrode for the ISF extraction.

### 2.5. Functional and Sensitive Modification of the Electrodes

**Figure 1:**
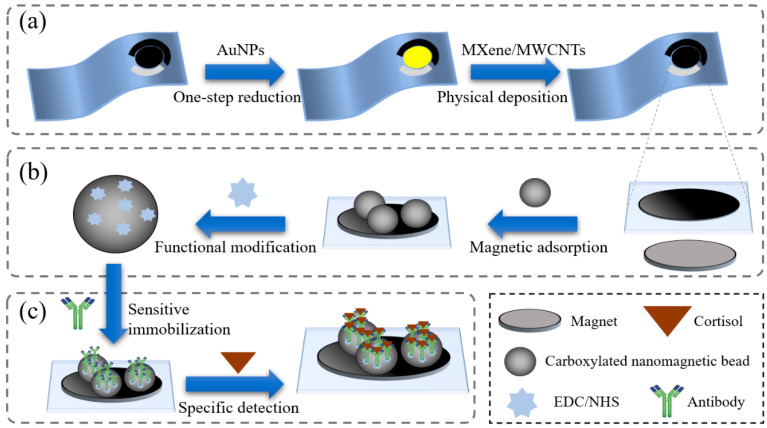
Schematic diagram of the electrode preparation. (**a**) Functional modification of the working electrodes. (**b**,**c**) Sensitive immobilization of the electrodes based on the carboxylated nanomagnetic beads.

Based on a one-step reduction, the AuNPs were functionally modified on the screen-printed electrodes to improve the electrochemical properties. After the aqueous solution of HAuCl_4_ (100 μL, 1%) was dripped ono the surface of the working electrode, with the stimulation of SWV, the AuNPs were electrodeposited onto the electrode. Afterwards, the nanocomposites of MXene and MWCNTs were coated onto the AuNPs layer through physical deposition to enhance the electron transfer rate of the electrodes (Figure 1a). Moreover, to enhance the stability of the electrodes, a Nafion solution (5 μL, 1%) was added, forming a proton exchange membrane. For the investigation of the improvements in the functions of the modified nanomaterials, electrochemical characterizations of the electrodes were performed with different modification ratios. Subsequently, nine electrodes were prepared and modified with different volume ratios of the MXene and MWCNTs mixed solution (ratios ranging from 4:1 to 1:7), and CV tests under 20 mM [Fe(CN)_6_]^3−/4−^ were performed for the optimization.

By adding a plane magnet under the electrode and with adjustments to the magnet positions, the carboxylated nanomagnetic beads could attach to the surface of the electrode stably and uniformly. Under the efforts of the EDC and NHS, the cortisol antibodies were immobilized onto the carboxylated nanomagnetic beads (Figure 1b,c). Afterwards, a BSA solution (10 μL, 1 mg/mL) was added to the nanomagnetic beads to block the nonspecific binding sites of the carboxyl. Then, the electrodes were washed with deionized water and dried naturally to complete the detections. Meanwhile, the optimal cortisol antibody concentration was investigated (from 1 μg/mL to 8 μg/mL), and the electrochemical characterizations of the sensitive modification were performed using CV tests.

### 2.6. Development of the Wearable Sensing System

**Figure 2:**
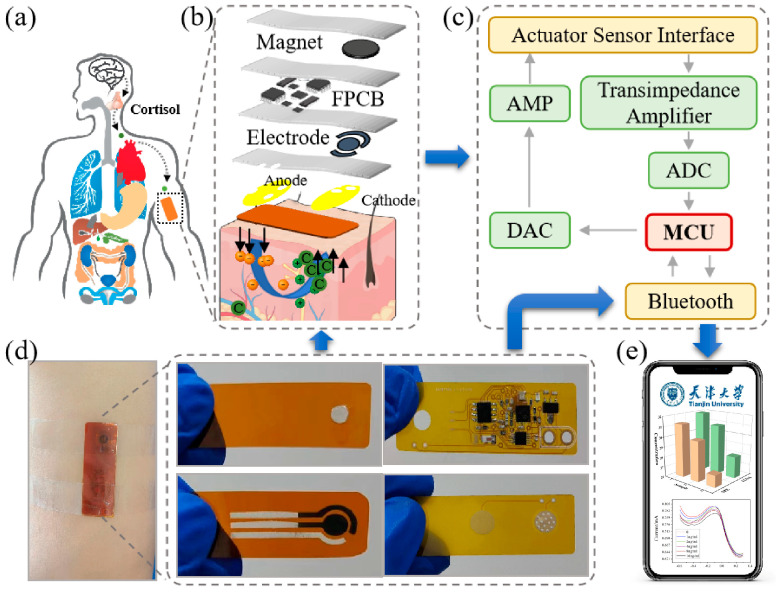
The non-invasive, wearable cortisol sensing system. (**a**) The secretion process of cortisol in the human body when exposed to external stimuli. (**b**) An explosion diagram of the sensing system. (**c**) The working principle of the sensing system. (**d**) An actual picture of the sensor and detection equipment. (**e**) A schematic diagram of the mobile terminal detection interface.

Flexible printed circuit techniques were utilized for the development of the wearable detection system. The secretion process of cortisol in the human body when exposed to external stimuli is shown in Figure 2a, and the explosion diagram of the wearable sensing system is shown in Figure 2b. For the non-invasive extraction of ISF and the rapid detection of cortisol, the electrochemical approaches of RI and DPV were integrated into the FPCB. This wearable detection system consisted of an electrochemical analog front-end, a microcontroller unit (MCU), Bluetooth, and a power supply (Figure 2c and Figures S2 and S3). The electrochemical analog front-end was designed for signal recognition, including one feedback amplifier and two high-gain span resistance amplifiers. The STM32 was selected as the MCU for the control of the analog front-end, exchanging data and commands. The Bluetooth module was used for communications between the wearable system and mobile devices, such as smartphones. The power supply provided stable energy to the detection system. Further, an independent ground layer was utilized to reduce the power impedance.

### 2.7. Replacement of the Sensing Elements and Detection in Real Samples

Since nanomagnetic beads were utilized for the self-assembly of the sensing elements, the replacement of sensing elements was performed and verified experimentally. Firstly, the nanomagnetic-beads-based wearable sensing system was utilized for the detection of cortisol levels ranging from 0 to 32 ng/mL. Afterwards, the sensitive immobilized nanomagnetic beads were released from the electrodes through removing the back magnet. Then, after being flushed with deionized water and dried at room temperature, the electrodes were re-modified with new sensitive immobilized nanomagnetic beads through the back magnet. Meanwhile, the distribution of nanomagnetic beads was adjusted uniformly through slightly shifting the position of the magnet. Then, the replacement of the sensing elements was completed, and the re-modified electrodes were utilized for the detection of cortisol with same situations for stability verification.

The proposed wearable sensing system was utilized for detection in real samples of human ISF under optimal conditions. Figure 2d shows an actual picture of the detection device, which consisted of a fixed layer, the FPCB, a detection layer, and an extraction layer. It was fixed on a volunteer’s arm with adhesive tape for detection. Figure 2e shows a schematic diagram of the mobile terminal detection interface, which was mainly used for real-time feedback of the detection results. The recruited volunteers were asked to watch a horror movie for 5 min while their ISFs were extracted at different stages of watching. These samples were divided into before watching, during watching, and after watching. Also, the temperature changes in the target’s skin were monitored with an infrared thermometer every 15 s while ISF extraction took place.

## 3. Results and Discussion

### 3.1. Optimization of the Materials for the Electrode Modification

**Figure 3:**
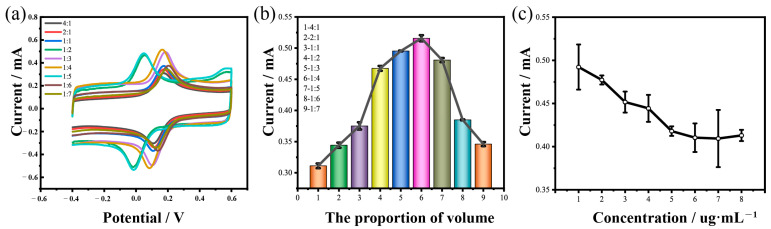
Optimization of the volume ratios of MXene and MWCNTs, and optimization of the antibody concentrations. (**a**) CVs of the MXene/MWCNTs with different volume ratios of MXene and MWCNTs. (**b**) Dependence of the current responses on the volume ratios of MXene and MWCNTs. (**c**) Dependence of the current responses on the concentrations of the cortisol antibody.

The CV tests on the nanocomposites were performed with different volume ratios of MXene and MWCNTs in [Fe(CN)_6_]^3+^/[Fe(CN)_6_]^4+^ (ranging from 4:1 to 1:7 for the optimization of nanocomposites preparation (Figure 3a)). As shown in Figure 3b, the current peak was first increased from 4:1 to 1:4 and then decreased from 1:4 to 1:7. Therefore, the optimized volume ratio for the MXene and MWCNTs was 1:4, which was selected for the experiments that followed.

The optimization of the sensing elements concentration was also performed, ranging from 1 μg/mL to 5 μg/mL. As illustrated in Figure 3c, the current peak decreased as the cortisol antibody concentration gradually increased due to the microsphere–antibody conjugation on the carboxylated nanomagnetic bead surfaces, leading an increase in the resistance of the electrode. However, the changes in the peak current trended toward stable while the cortisol antibody concentration reached 5 μg/mL. This indicated that the cortisol antibody was bound at the surface of the carboxylated nanomagnetic beads and had reached saturation. Therefore, the most efficacious cortisol antibody concentration was 5 µg/mL, which was utilized for the experiments that followed.

### 3.2. Morphological and Electrochemical Characterizations

**Figure 4:**
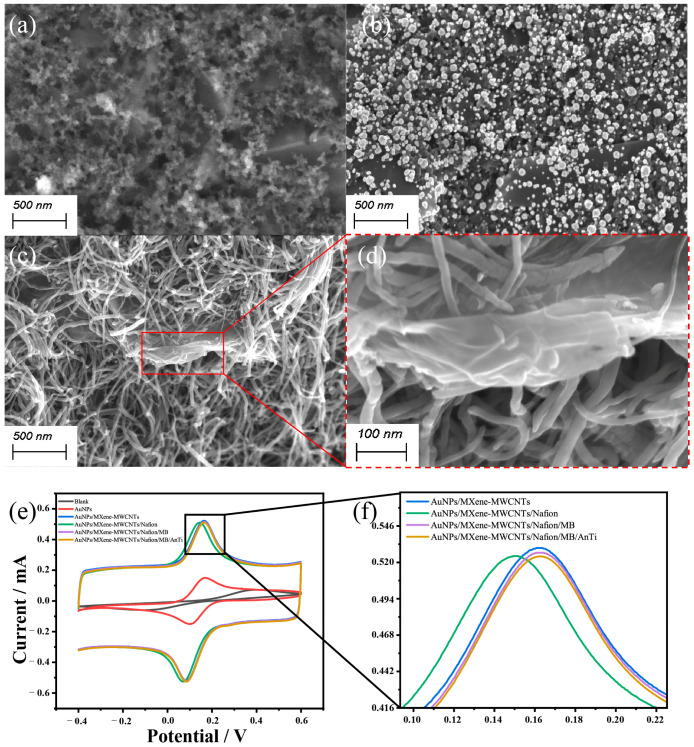
Characterizations of the electrodes with the different modifications. (**a**–**d**) SEM images of the SPE, AuNPs/SPE, and MXene-MWCNTs/SPE. (**e**,**f**) CVs of the SPE at each modification stage. MB, nanomagnetic beads; AnTi, antibody.

Gold nanoparticles are widely used in working electrode surface modifications due to their relatively large surface area, chemical stability, and biocompatibility. As shown in Figure 4a,b, it could be concluded that a layer of AuNPs was deposited on the surface of the electrode. In addition, by comparing the redox peak currents of the SPE and the AuNPs-modified SPE (AuNPs/SPE), it could be observed that the redox peak currents were significantly increased after the modification of the AuNPs (Figure 4e). The decreased peak-to-peak separation of the AuNPs/SPE also demonstrated that the AuNPs had been successfully modified onto the surface of the working electrode and that the AuNPs remarkably enhanced the electrochemical conductivity. At the same time, the large specific surface area of the AuNPs increased the contact area between the WE and the MXene–MWCNTs mixed solution, making it easier for the deposition of the MXene and MWCNTs composites.

MXene is a type of two-dimensional inorganic compound that possesses excellent electrical conductivity, a large specific surface area, and good biocompatibility. Through the morphological characterization, it appeared as brick or mortar with a lamellar shape (Figure 4c,d). As shown in Figure S4, compared to the AuNPs/SPE, the increase in the peak current of the redox peaks for the AuNPs/MXene/SPE was not significant, and the detailed reasons are discussed below. Firstly, individual modification of the MXene could not ensure a firm deposition, and some MXene may have been washed out when liquid added. Secondly, the electrode required time to dry after being modified with MXene, during which the exposed MXene directly contacted oxygen and became oxidized. Thirdly, a single MXene film lacks interlayer interactions, and this resulted in mechanical brittleness and made it prone to fracture under external forces. To address these shortcomings, the nanocomposites of MWCNTs and MXene were synthesized and utilized for the modification of the electrodes.

As shown in Figure S4, the redox peak current of the AuNPs/MXene-MWCNTs/SPE was significantly higher than those of the AuNPs/MXene/SPE and AuNPs/MWCNTs/SPE, and the current was approximately six times higher than that of bare SPE. That was because the nanocomposites could disperse stress through the MWCNTs, reducing the formation and propagation of cracks when they were subjected to external forces and thereby enhanced their mechanical strength and toughness. In addition, the MWCNTs behaved like glue, which could make the binding of the MXene more stable, and it prevented the detachment of the MXene. Furthermore, the addition of the MWCNTs could provide additional conductive pathways, reducing the aggregation of MXene and enhancing the overall electrical conductivity. Compared with the AuNPs/SPE, the AuNPs/MXene-MWCNTs/SPE exhibited an obvious increase in the peak current for the redox probe, which could be attributed to the modification of the MXene and MWCNTs, which further enhanced the active surface area of the working electrode. At the same time, the decreased peak-to-peak separation of the AuNPs/MXene-MWCNTs/SPE also demonstrated that the AuNPs and the mixed material of the MXene-MWCNTs remarkably enhanced the electrochemical conductivity of the electrodes. This indicated that the composite nanomaterials could improve the conductivity of the electrodes, providing ideal conditions for cortisol detection. After the addition of the Nafion, there was a slight decrease in the peak current of the working electrode, which indicated the protection provided by the Nafion to the electrode and the hindrance of the electron transfers.

As shown in Figure S5, with the immobilization of the nanomagnetic beads, the redox peak currents of the functional modified electrodes increased again. This was because the nanomagnetic beads were composed of iron oxide (Fe_3_O_4_). The electrons in the Fe_3_O_4_ were rapidly transferred between the two oxidation states of iron, resulting in excellent electrical conductivity in the solid state. In general, the external surfaces of the nanomagnetic beads were modified with the charged functional groups, which enhanced the conductivity of the working electrode. Afterwards, the current response of the redox peak was reduced after the sensitive immobilization of the antibodies, as the antibodies formed covalent bonds with the functional groups, hindering the electron transfer. This also demonstrated that the nanomagnetic beads had successfully bonded with the antibody. Further, the influence of the Nafion, nanomagnetic beads, and antibodies on the electrochemical performance was negligible.

### 3.3. Evaluation of the Cortisol-Sensing Performance

**Figure 5:**
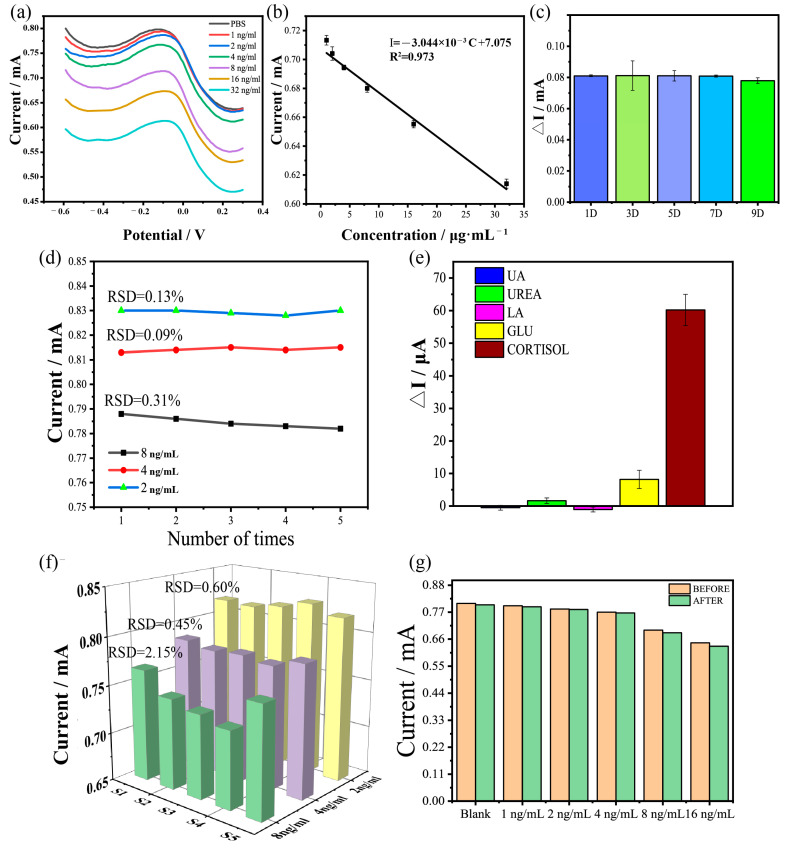
Electrochemical performance of the modified electrodes. (**a**) DPV response for the different concentrations of cortisol (1−32 ng/mL). (**b**) Linear relationship between the current changes and cortisol concentrations. (**c**) Stability of the modified electrodes for 9 days. (**d**) Repeatability of the modified electrodes for cortisol detection at 2 ng/mL, 4 ng/mL, and 8 ng/mL. (**e**) Selectivity of the electrodes among the UA (4 ng/mL), urea (4 ng/mL), LA (4 ng/mL), glucose (4 ng/mL), and cortisol (4 ng/mL). (**f**) Reproducibility with five electrodes for the detection of 2 ng/mL, 4 ng/mL, and 8 ng/mL of cortisol. (**g**) DPV response changes for the electrodes before and after the replacement of the sensing elements using nanomagnetic beads.

In order to evaluate the performance of our sensing system, cortisol solutions with concentrations that ranged from 1 ng/mL to 16 ng/mL were tested based on the electrochemical approach DPV. As shown in Figure 5a, the peak current value for cortisol decreased as the concentration of cortisol increased. This could have been due to the formation of an insulating immune complex after the binding of the carboxylated nanomagnetic beads and the antibody, which prevented electron transfers and increased the impedance of the electrodes. Thus, the peak currents for the different concentrations of cortisol were analyzed and fitted linearly (Figure 5b). The calculation formulas used were:

(1) I=K*C+i and

(2) $$R^{2}=\frac{SSR}{SST}=\frac{\sum {\left({\overset{\wedge }{i}}_{x}-\bar{i}\right)}^{2}}{\sum {\left(i_{x}-\bar{i}\right)}^{2}},$$

where C was the actual concentration, i was the actual peak current, $\overset{\wedge }{i}$ was the expected peak current, and $\bar{i}$ was the average peak current. The regression equation was $I=-3.044\times 10^{-3}C+7.075$, with a correlation coefficient of 0.973. The fitting equation was linear, and changes in the peak current directly related to changes in the concentration of cortisol. Therefore, this sensing system was capable of distinguishing fine changes in cortisol concentrations that ranged from 0 to 32 ng/mL. The electrodes modified with the functional materials and the sensing elements were prepared and stored at 4 °C before each test. To evaluate the stability, cortisol with a concentration of 4 ng/mL was measured on days 1, 3, 5, 7, and 9 (Figure 5c). The peak current varying between tests was negligible, indicating that the electrode possessed high stability. In addition, we independently prepared electrodes for the detection of cortisol with concentrations of 2 ng/mL, 4 ng/mL, and 8 ng/mL, from which the relative standard deviations (RSDs) were found to be 0.13%, 0.09%, and 0.31%, respectively, indicating that the repeatability of our fabricated electrodes was stable enough for cortisol detection (Figure 5d).

### 3.4. Anti-Interference Analysis of the Sensing System

For the evaluation of the sensing specificity, UA, urea, LA, and glucose, which were present in the real ISF samples, were measured using this nanomagnetic-beads-based sensing system. The concentrations of all these analytes were set at 4 ng/mL, and all the experimental conditions were the same. The differences in the DPV responses are shown in Figure 5e, where the different signal responses of the interferences showed negligible changes. This was because when the cortisol antibodies were bound to the nanomagnetic beads, they could be considered as a new type of nanomaterial, with functions for detecting biological molecules. An antigen–antibody complex was formed when cortisol bound to a cortisol antibody. This complex had a lack of conductivity, which led to a decrease in the conductivity of this new nanomaterial, and so the conductivity of the working electrodes also decreased. The result was that the peak current of DPV decreased as the concentration of cortisol increased. Furthermore, the MXene, MWCNTs, and Nafion did not have functional groups on their surfaces, which meant they could not react or bind with any biological molecules. Due to the extremely high specificity of the cortisol antibodies, they would not bind with any biological molecules other than cortisol. Therefore, this sensing system would not be interfered with by other substances, and it offered a high selectivity towards cortisol.

### 3.5. Reproducibility and Rapid Replacement of the Sensing Elements

An additional five electrodes with functional and sensitive modifications were prepared independently for the evaluation of reproducibility, and they were named S1 to S5. Cortisol solutions with concentrations of 2 ng/mL, 4 ng/mL, and 8ng/mL were tested by our sensing system using S1 to S5. The results are shown in Figure 5f, where it can be seen that the response of a single electrode for the same cortisol concentration was similar. The results were categorized into three groups based on the concentrations of cortisol and the RSDs of each group, and the RSDs were found to be 2.15%, 0.45%, and 0.60%, respectively. The values of the RSDs for each group were very small, which indicated that the measurements had excellent accuracy and reliability. Furthermore, the responses of the electrodes based on the nanomagnetic beads and cortisol antibodies were nearly consistent, suggesting that the fabrication of the sensing electrodes was reproducible.

In Figure 5g and Figure S6, it can be observed that the electrode continued to detect cortisol before and after the sensing elements were replaced, and the responses maintained good correlations between the peak currents and the cortisol concentrations. Additionally, by comparing the detection results before and after the replacement of the sensing elements, it was found that the peak currents of the electrodes to same concentrations of cortisol were highly consistent. This indicated that the replacement of the sensing elements had virtually no impact on the detection capabilities. This could be attributed to the advantages of the Nafion and the nanomagnetic beads. Firstly, Nafion possesses excellent heat resistance and corrosion resistance [33]. A Nafion solution can form a layer of film with superior stability when it is applied to the surface of a nanomaterial. The Nafion film can prevent deformation of the nanomaterials caused by changes in the temperature of working electrodes and long-term exposure to liquids, extending the service life of the electrodes. Secondly, nanomagnetic beads can be pre-coupled with antibodies and stored as reserve-sensing elements. Due to the sub-micron structures of nanomagnetic beads, they can be carried in large quantities. Also, nanomagnetic beads can be quickly fixed and replaced under the control of a magnetic field due to their superparamagnetic and rapid magnetic responsiveness [34]. Therefore, the rapid replacement of sensing elements was achieved. Furthermore, it was proven that the electrode could update or replace the sensing elements according to actual detection needs without affecting the functional modification state and detection ability of the working electrode. This also addressed the issues of low repeatability, low flexibility, and an inability to perform multiple measurements associated with traditional biosensors.

### 3.6. Extraction of Interstitial Fluid and the Evaluation of Mental Health

**Figure 6:**
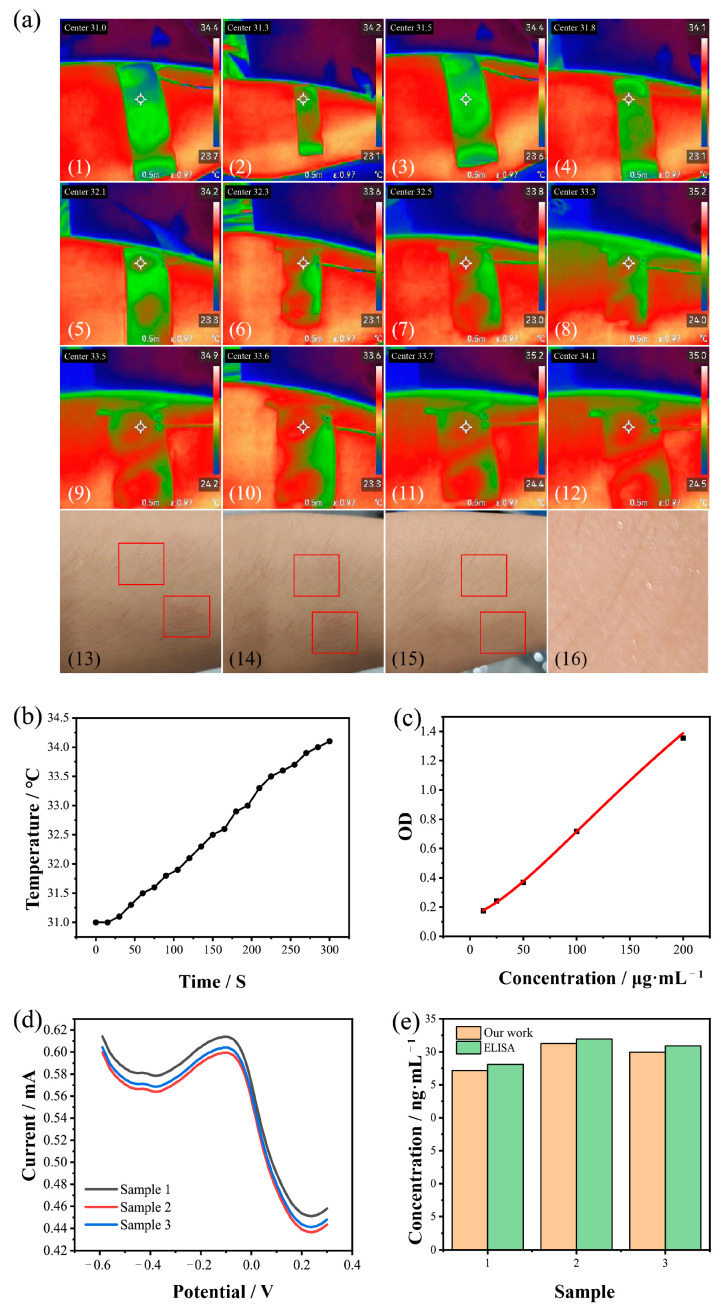
The ISF extraction and real sample detection. (**a**) Near-infrared images of the analysis of skin temperature. (**b**) Trends in the temperature changes during the extraction of ISF. (**c**) The linear relationship between the OD and the cortisol concentration for the ELISA. (**d**) DPV responses based on our sensing system for the real samples. (**e**) Comparison of the real sample evaluations between our work and the commercial ELISA tests. OD, optical density.

As we can see in Figure 6a,b and Figure S7, throughout the experiments, the temperature of the extraction electrode gradually increased from an initial 31 °C to 34.1 °C (at room temperature (25 °C)). The temperature increase was relatively slow, and the change was minimal. After the electrode was removed, a slight reddening on the part of the volunteer’s arm that was in direct contact with the extraction electrode could be observed. However, after a period of observation, we found that the redness gradually faded, and within 10 min, the redness had completely disappeared. The volunteer did not experience any discomfort throughout the process. At the same time, we could observe that when the volunteer removed the electrode, there was still liquid present on the area covered by the extraction electrode, which explained that the extraction electrode was functioning normally and the ISF had been successfully extracted.

While the recruited volunteers were watching a horror movie, ISF samples were extracted and divided into the following three stages: before watching, during watching, and after watching. We designated the sample extracted before watching as sample 1, the sample during watching as sample 2, and the sample after watching as sample 3. Then, we analyzed the concentrations of cortisol in the collected samples using ELISA, and we fitted the standard optical density and concentrations (Figure 6c). The concentrations of cortisol in these samples were calculated according to the fitted curve, and the calculated concentrations were as follows: sample 1, 28.12 ng/mL; sample 2, 31.95 ng/mL; and sample 3, 30.90 ng/mL. Then, these three samples were also tested using our sensing system, and DPV peak currents were obtained (Figure 6d). The increase in cortisol concentration in sample 2 may have been due to the scary scenes in the movie, which stimulated the volunteers, leading to the release of cortisol [6,7,10]. The decrease in cortisol concentration in sample 3 may have been related to the volunteers’ adaptation to the scary scenes. The stimulation of the volunteers due to the movie decreased or disappeared, causing the body’s cortisol release to return back to a normal level; therefore, the cortisol concentration in sample 3 was lower than that in sample 2. However, since cortisol cannot be metabolized in a short time, the cortisol concentration in sample 2 was still higher than that in sample 1. This can be also utilized for the explanation of the changes in the concentrations among these three samples, which were relatively small. Afterwards, based on the goodness of the fitting curve and the measured DPV peak currents, the results of the real samples for the three stages were detected as 27.16 ng/mL, 31.25 ng/mL, and 29.95 ng/mL, respectively. Based on a comparison of the two sets, the differences in the results for our work and the ELISA were approximately 1 ng/mL, which showed good stability and accuracy (Figure 6e and Figure S8). Therefore, the difference was negligible. These results demonstrated that the detection approach was reliable for the evaluation of cortisol in human ISF.

### 3.7. Advantages of Mental Health Evaluation and Future Improvements

In this study, an electrochemical immunosensor was proposed for the detection of cortisol in interstitial fluid for the evaluation of mental health. Firstly, by combining with reverse iontophoresis technology, the sensor was able to actively and non-invasively extract interstitial fluid under any circumstances [25,26]. Secondly, we fully utilized the excellent carrying capacity and superparamagnetic and rapid magnetic responsiveness of nanomagnetic beads [34]. On one hand, this approach increased the number of antibodies that the sensor could bind to, thereby enhancing the sensor’s detection limits. On the other hand, the nanomagnetic beads were applied as carriers for the antibodies, allowing the for the preparation and carrying of the sensing elements in advance, which could be rapidly replaced under the control of a magnetic field. Therefore, the difficulty in replacing sensing elements in traditional electrochemical immunosensors has been solved.

## 4. Conclusions

In summary, a non-invasive, wearable electrochemical biosensor is reported herein for the detection of cortisol in human ISF for the evaluation of mental health. Based on a one-step reduction in nanocomposites, the electron transfer rate and conductivity of the electrodes were enhanced. With the self-assembly property of a magnet, this nanomagnetic-beads-based sensing system has solved the difficulty of replacing sensing elements in traditional electrochemical biosensors. By integrating a reverse iontophoresis technique on the FPCB, ISF could be extracted conveniently for rapid analysis. Combined with the positive correlation between cortisol concentration and mental stress, the mental stress of emergency rescuers can be evaluated, which can not only provide feedback on the psychological statuses of rescuers but also effectively improve rescuer safety and rescue efficiency.

## Data Availability

The data are available on request.

## Supplementary Materials

The following supporting information can be downloaded at: https://www.mdpi.com/article/10.3390/bios15030140/s1, Figure S1. Design of the electrodes. (a) The design of screen-printed electrode. (b,c) The front and back views of the extraction electrode; Figure S2. The front (a) and back (b) views of the printed circuit board of wearable sensing system.; Figure S3. Circuit schematic diagram of the wearable sensing system for cortisol detection; Figure S4. Cyclic voltammetry characterization of electrodes with different modifications; Figure S5. EIS plots of different electrodes; Figure S6. Linear relation between current changes and cortisol concentrations before and after the replacement of sensing element; Figure S7. Temperature changes for every 15 s during the extraction of interstitial fluid by reverse iontophoresis; Figure S8. (a) The standard OD value and concentration data of ELISA. (b) The OD value and concentration of real samples in ELISA, the response current and concentration of real samples in electrochemical immunosensor.
