# Supplementary material for: Rapid Mental Stress Evaluation Based on Non-Invasive, Wearable Cortisol Detection with the Self-Assembly of Nanomagnetic Beads
Source: Biosensors (Basel). 2025 Feb 23;15(3):140. doi: 10.3390/bios15030140 (PMC11940475; doi:10.3390/bios15030140)
Supplement: Supplementary file 1 [file biosensors-15-00140-s001.zip › biosensors-3446960-supplementary.pdf]

Supplementary Materials

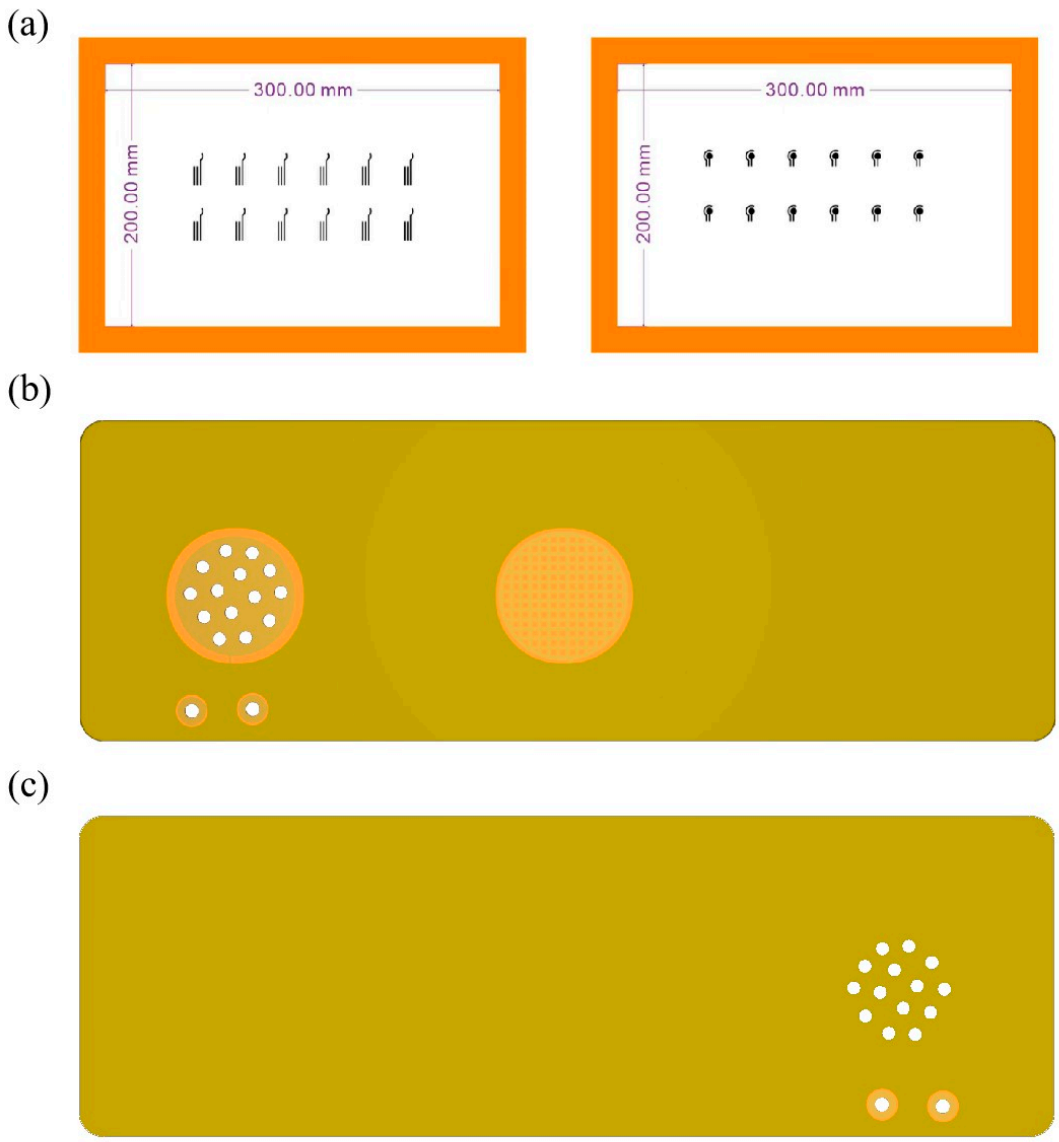

Figure S1. Design of the electrodes. (a) The design of screen-printed electrode. (b-c) The front and back views of the extraction electrode.

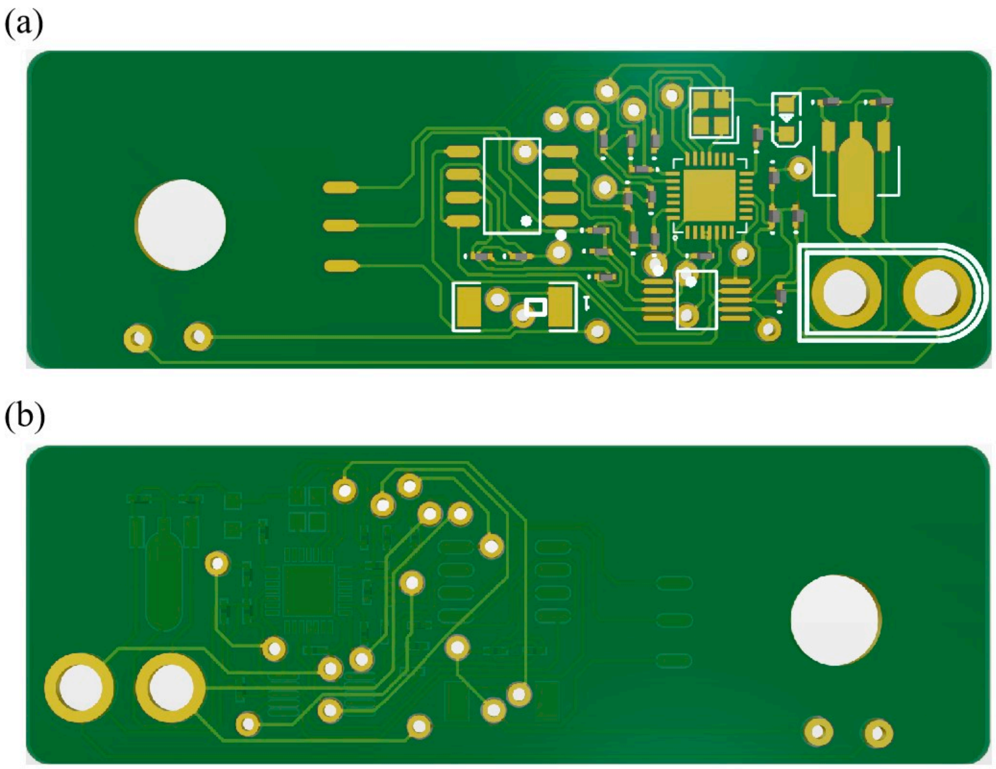

Figure S2. The front (a) and back (b) views of the printed circuit board of wearable sensing system.

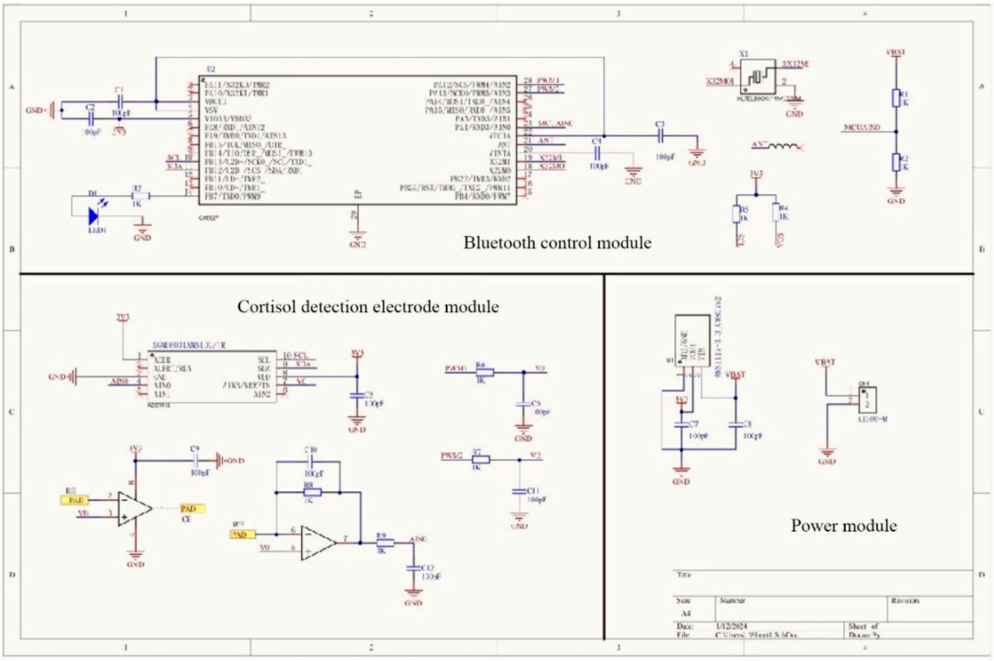

Figure S3. Circuit schematic diagram of the wearable sensing system for cortisol detection.

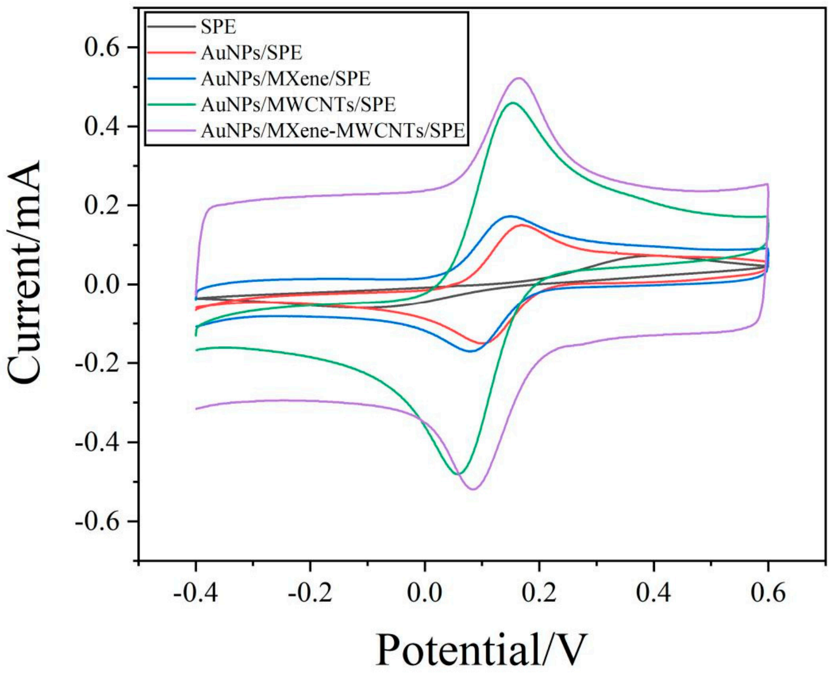

Figure S4. Cyclic voltammetry characterization of electrodes with different modifications.

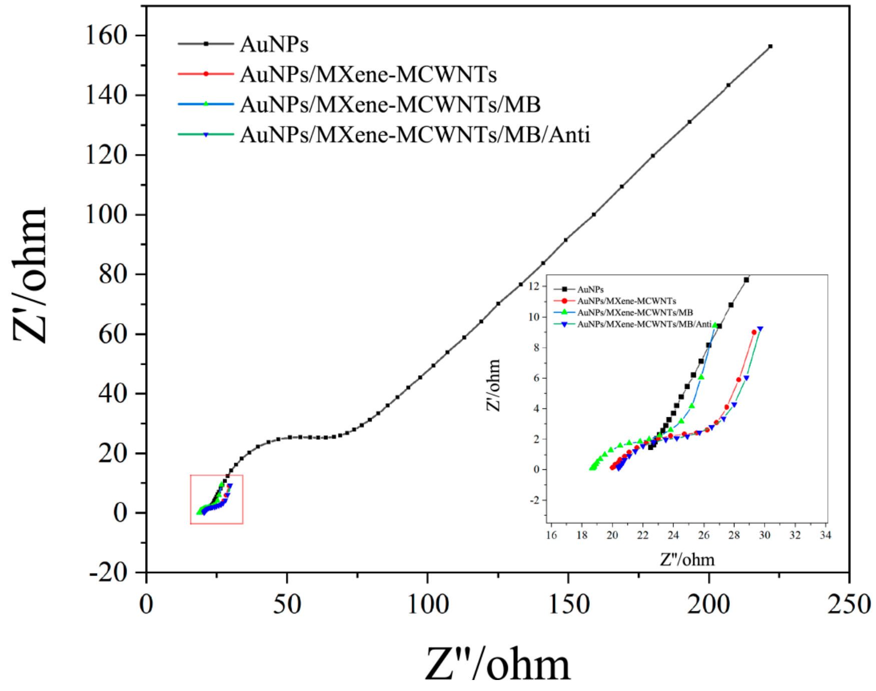

Figure S5. EIS plots of different electrodes.

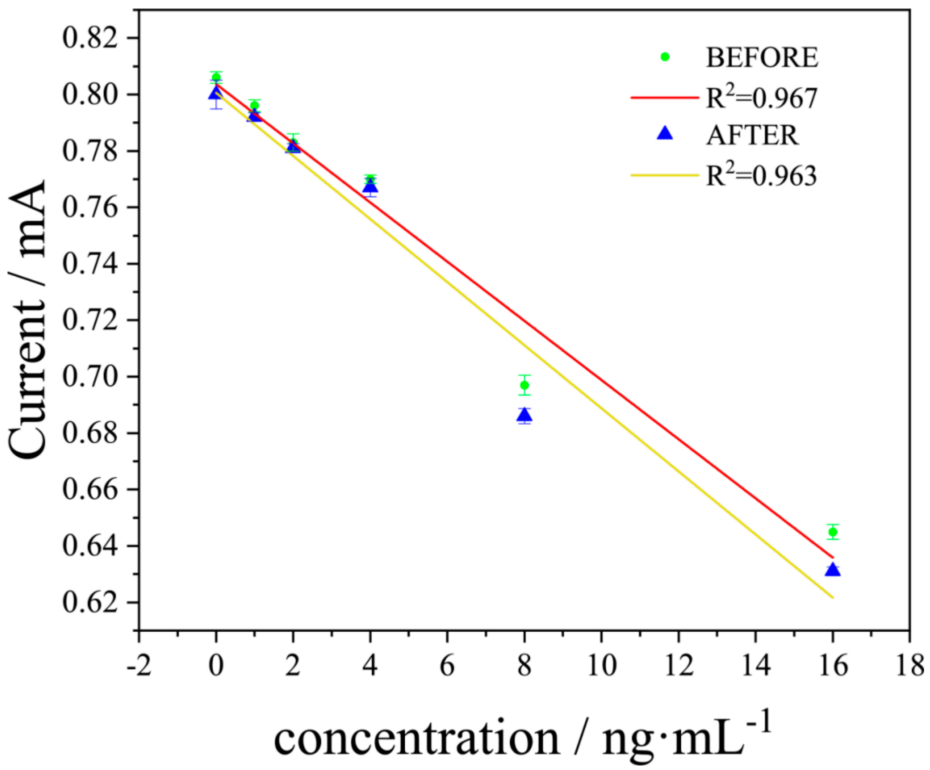

Figure S6. Linear relation between current changes and cortisol concentrations before and after the replacement of sensing element.

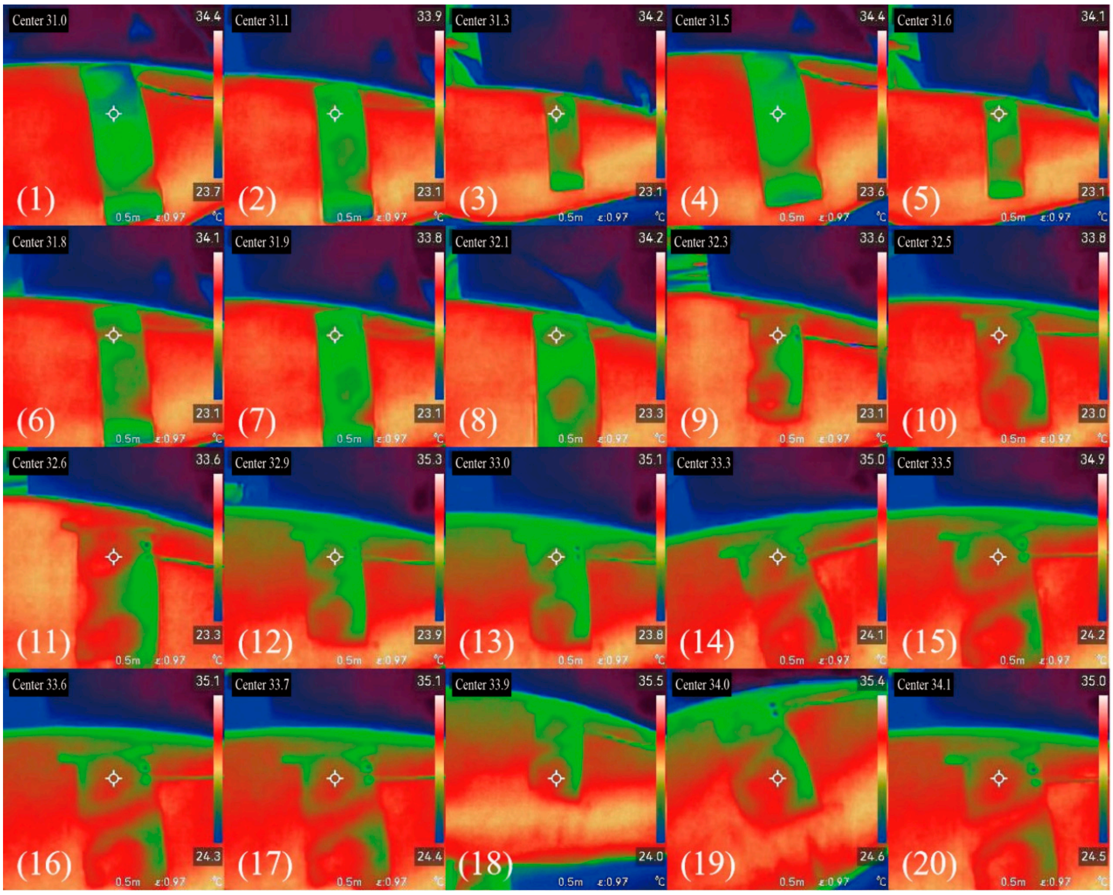

Figure S7. Temperature changes for every 15 seconds during the extraction of interstitial fluid by reverse iontophoresis.

(a)

Standard	1	2	3	4	5
A	0.173	0.242	0.369	0.717	1.345
B	0.174	0.242	0.37	0.716	1.357
C	0.174	0.243	0.37	0.717	1.36
AVERAGE	0.173666667	0.242333333	0.369666667	0.716666667	1.354
Error bar	0.000471405	0.000471405	0.000471405	0.000471405	0.006480741
Concentration (ng/ml)	12. 5	25	50	100	200

(b)

Sample	1	3	6
A	0.268	0.249	0.267
B	0.27	0.249	0.262
C	0.27	0.249	0.262
AVERAGE	0.269333333	0.249	0.263666667
Concentration of ELISA (ng/ml)	31. 95386	28. 11574	30. 90195
Error bar	0.000942809	0	0.002357023
DPV/Current	0.0005995	0.000614	0.0006041
Concentration of Biosensor (ng/ml)	31. 25402	27. 16446	29. 95664

Figure S8. (a) The standard OD value and concentration data of ELISA. (b) The OD value and concentration of real samples in ELISA, the response current and concentration of real samples in electrochemical immunosensor.
